# Vitamin D: A Nutraceutical Supplement at the Crossroad Between Respiratory Infections and COVID-19

**DOI:** 10.3390/ijms26062550

**Published:** 2025-03-12

**Authors:** Manuela Rizzi, Pier Paolo Sainaghi

**Affiliations:** 1 Department of Health Sciences (DiSS), Università del Piemonte Orientale (UPO), 28100 Novara, Italy; 2 IRCAD (Interdisciplinary Research Center of Autoimmune Diseases), Università del Piemonte Orientale (UPO), 28100 Novara, Italy; 3 Department of Translational Medicine (DiMeT), Università del Piemonte Orientale (UPO), 28100 Novara, Italy

**Keywords:** vitamin D, COVID-19, SARS-CoV-2, respiratory infections, clinical trials, immunomodulation

## Abstract

Even though in mid-2023 the World Health Organization declared the end of the public health emergency of international concern status for COVID-19, many areas of uncertainty about SARS-CoV-2 infection pathophysiology remain. Although in the last 4 years pharmaceutical industries widely invested in the development of effective antiviral treatments and vaccines, large disparities in their availability worldwide still exist, thus fostering the investigation of nutritional supplements as adjuvant therapeutic approaches for disease management, especially in resource-limited settings. During the COVID-19 pandemic, vitamin D has been widely used as an over-the-counter solution to improve disease evolution, thanks to its known immunomodulatory and anti-inflammatory actions. Ecological and observational studies support a relationship between hypovitaminosis D and COVID-19 negative outcomes and, according to this evidence, several research groups investigated the role of vitamin D supplementation in protecting from SARS-CoV-2 infection and/or improving disease evolution. This narrative review is intended to offer insights into the existing data on vitamin D’s biological effects in respiratory infections, especially in COVID-19. Furthermore, it will also offer a brief overview of the complex interplay between vitamin D and vaccine-elicited immune response, with special attention to anti-COVID-19 vaccines.

## 1. Introduction

At the end of 2019, a cluster of pneumonia cases of unknown origin was first described in Wuhan (China), representing the beginning of one of the deadliest pandemics in the world’s history. Early in the pandemic course, the etiological agent of the coronavirus disease 2019 (COVID-19) was identified as the severe acute respiratory syndrome coronavirus 2 (SARS-CoV-2). SARS-CoV-2 is an enveloped, single-stranded, RNA virus from the Coronaviridae family, characterized by a high rate of mutation, accounting for the different viral strains being dominant during the consecutive waves of disease outbreaks [1,2,3].

After more than 4 years since the first patient identification, it is well accepted that COVID-19 is a very heterogeneous disease, whose clinical manifestations could range from an asymptomatic or paucisymptomatic manifestation to severe and life-threatening conditions, mainly characterized by interstitial pneumonia, acute respiratory distress syndrome (ARDS), hypercytokinemia associated to a dysregulated immune response, impaired coagulation, and severe multiorgan failure [3,4,5,6,7,8].

Due to the high socioeconomic impact of the COVID-19 pandemic, since the beginning of the emergency huge efforts have been made to develop effective and selective treatments as well as vaccines allowing preventive immunization of the global population. To date, several types of vaccines have been developed, and those based on mRNA technology (i.e., Pfizer–BioNTech BNT132b2 and Moderna mRNA-1273 vaccines) were the most commonly used to sustain different immunization campaigns worldwide [9,10,11,12]. Moreover, a large number of pharmacological agents have been evaluated in terms of anti-SARS-CoV-2 activity, such as monoclonal antibodies, immunomodulators (i.e., drugs targeting the main players of the COVID-19-associated cytokine storm), and repurposed as well as newly developed antivirals, finally resulting in several preclinical and clinical studies, allowing the emergency use authorization for many of them [3,10,13].

In spite of this immense effort of the pharmaceutical industry, at the time of writing, only a part of the world population is fully vaccinated, and only a few specific anti-SARS-CoV-2 antiviral agents have been marketed. Moreover, these drugs showed a limited treatment window, with different efficacy depending on the phase of the infection at the time of pharmacological treatment, as well as on the circulating viral variant [3,9,10,14].

Considering that newly emerging variants are characterized by increased transmissibility associated with an increased ability to escape from natural and vaccine-induced immunity and by a reduced response to some pharmacological treatments, it is clear that SARS-CoV-2 management is still challenging. To date, effective therapeutic options to manage the clinical manifestations of the disease are still limited: only a few anti-COVID-19 drugs, such as nirmatrelvir+ritonavir (Paxlovid) and molnupiravir, are available for outpatient management, while the majority of the targeted pharmacological approaches are generally available only in hospital context (i.e., remdesivir, corticosteroids, tocilizumab, baricitinib, anakinra), thus limiting their use in outpatient and resource-limited settings [4,9,10,13,14].

In this scenario, it is not surprising that over-the-counter solutions, as well as traditional remedies coming from traditional Chinese and Indian medicine, still raise interest both in the general population and in the scientific community, as they can represent add-on interventions intended to prevent and/or mitigate COVID-19 clinical manifestations [13,15,16,17,18,19,20].

It is well known that a balanced diet is essential for human well-being, as malnourished individuals are more prone to infectious diseases [7,21,22,23], especially taking into consideration that malnutrition hardly affects immune function, as immune cells rely on nutritive substrate availability to quickly respond to noxious threats [7,21,22,24,25,26]. Among the most important nutrients known to play an important role in assuring immune system efficiency, there are vitamins (i.e., vitamin A, vitamin C, and vitamin D), as well as oligoelements (i.e., zinc, selenium), prebiotics and probiotics [15,21,26,27,28,29].

Even though, especially during the initial phases of the pandemic, several clinical trials and observational studies have focused on nutritional supplementation to identify a potential supportive approach able to prevent or mitigate COVID-19 outcomes, the obtained results were inconclusive, due to the large heterogeneity in the study design, as well as, in some cases, to the small number of patients involved, thus preventing the definition of a clear consensus around their clinical use [21,30]. Among the over-the-counter solutions that were investigated with conflicting results (summarized in Table 1), the only one that underwent a great in-depth investigation was represented by vitamin D, a chemical compound showing a wide range of biological activities, among which the immunomodulatory one seems to be strongly related, not only to immune protection in general, but also specifically to respiratory system protection against pathogen invasion.

This narrative review summarizes the available evidence about vitamin D’s biological effects on respiratory infections, especially in COVID-19 (considering both disease evolution and vaccine-elicited immune responses). A literature search was conducted by screening the PubMed, Scopus, and Google Scholar repositories up to 15 December 2024 using the following keywords “vitamin D”, “COVID-19”, “SARS-CoV-2”, “respiratory infections”, “nutraceuticals”, “dietary supplements”, “respiratory system”, “immune function”, and “vaccination response”, alone or in combination. In addition, to avoid missing relevant papers focused on the topic, the references of the original articles, as well as the related results suggested by the different search engines were also considered. Furthermore, when the bibliographic search resulted in an extensive quantity of results, the selection was limited to clinical trials and to the most recent clinical results. Only papers published in English, French, and Spanish with complete full text available were considered.

## 2. Vitamin D: A Natural Compound with Pleiotropic Actions in the Human Body

Vitamin D has long been known as an essential nutrient in all vertebrates, where it is necessary to maintain calcium and phosphorus homeostasis. In the last decades, several studies highlighted that such compound exerts also several extra-skeletal functions, thus representing a key element in assuring whole-body well-being [4,43,44].

Humans can obtain the vitamin D they need from different sources: this lipophilic vitamin is naturally present in some foods, can be added artificially to some others (the so-called vitamin D fortified foods, such as infant milk), is available as an over-the-counter nutritional supplement and, among all, it can be directly synthesized by the human organism. In particular, skin exposure to sunlight represents the main source of vitamin D production in humans, accounting for nearly 80–90% of the vitamin D needs coverage, while dietary intake covers the remaining 10–20% [4,43,45,46].

At the skin level, UVB radiation promotes the conversion of 7-dehydrocholesterol to cholecalciferol, an inactive precursor that needs to undergo two subsequent hydroxylation steps to become biologically active. The first hydroxylation step, catalyzed by the CYP27A1 enzyme, occurs in the liver and results in 25-hydroxyvitamin D (also known as calcidiol), which is the main circulating form of vitamin D and, thanks to its half-life of approximately two weeks, represents the most commonly evaluated marker to assess vitamin D status. The second hydroxylation step, catalyzed by the CYP27B1 enzyme, occurs in renal tubular cells and results in 1,25-dihydroxyvitamin D (also known as calcitriol), the biologically active form of vitamin D [4,43,44,45,46,47,48].

Once released in the bloodstream at the renal level, the biologically active form of vitamin D functions as a steroid hormone and can interact with specific vitamin D receptors (VDRs). These VDRs could have different cellular locations, accounting for different biological responses following their activation. Some VDRs (nuclear VDR) are located inside the cell: after binding to vitamin D, they form a complex with the retinoid X acid receptor (RXR) which finally binds vitamin D response elements (VDREs). Nuclear VDR thus mediates genomic responses, by regulating, in a direct or indirect way, roughly 3% of the human genome, finally resulting in biological effects ranging from calcium and phosphorous homeostasis maintenance to energy metabolism and immune function control [43,47,49,50]. Instead, other VDRs are located on the plasma membrane (membrane VDR): after binding to vitamin D, these receptors interact with other membrane-resident proteins, finally resulting in the generation of secondary messengers (e.g., cAMP, phosphatidylinositol 3,4,5 triphosphate, Ca^++^) and in the activation of several downstream signaling pathways (e.g., PKA, PKC, MAPK) [49,51,52].

Considering the wide role of vitamin D in assuring human well-being, it has been estimated that it should be present in a sufficient concentration, corresponding to at least 20–30 ng/mL, while blood concentrations below 20 ng/mL indicate hypovitaminosis or a deficiency state. Since vitamin D levels below the sufficiency range (aka hypovitaminosis D) are associated with several negative health effects, affecting not only the musculoskeletal system, but also the immune domain, it is not surprising that several international agencies, such as the WHO, issued recommendations about the daily doses of vitamin D needed by different population groups [43,50,53].

Despite such efforts to ensure sufficient vitamin D intake, suboptimal levels of this nutrient still affect over 50% of the world population, regardless of age and ethnicity, thus representing a significant health problem worldwide [43,48,54]. To date, more than one billion individuals suffer to various extents of hypovitaminosis D, which clinically manifests as vitamin D insufficiency (when serum vitamin D levels range between 12 and 20 ng/mL) and vitamin D deficiency (when serum vitamin D levels are <12 ng/mL) [55,56,57]. In addition, it is worth noting that the world population is progressively aging, and the elderly represent a population at increased risk of hypovitaminosis D, due to a decrease in their skin’s ability to synthesize it, as well as to a poor dietary intake [43,46]. According to epidemiological data highlighting vitamin D deficiency prevalence on a global scale, it is thus not surprising the growing interest in vitamin D’s role in promoting the maintenance of healthy conditions, as it is recognized that vitamin D deficiency is positively associated with impaired immunity (e.g., autoimmune diseases manifestations, increased susceptibility to infections, poor outcomes from infectious diseases) as well as to a higher susceptibility for all-cause mortality [43,44,47,48,50,53,58,59,60,61].

## 3. Vitamin D and Immune Function

Biologically active vitamin D (1,25(OH)_2_D) is known to exert important immunomodulatory actions. All immune cell populations are known to express both the 1α-hydroxylase CYP27B1 and the vitamin D receptor (VDR), so their functions depend on their own ability to synthesize it. Locally produced vitamin D will thus mediate both genomic (e.g., gene transcription regulation following vitamin D-VDR complex translocation to the nucleus) and non-genomic (e.g., autocrine and paracrine signaling pathways activation, strengthening of epithelial and endothelial gap junction complexes) responses [43,47,58]. Furthermore, vitamin D’s immunomodulatory actions rely on its ability to down-regulate inflammatory responses, by suppressing pro-inflammatory cytokines production and by reducing oxidative stress, while increasing anti-microbial peptides (e.g., cathelicidin, β-defensin 2) and neutralizing antibodies production [44,46,47,53,58,62,63,64,65,66]. Moreover, vitamin D supports the host’s ability to fight infections by activating T and B lymphocytes and macrophages, which represent important immune cell populations actively involved in neutralizing infections [43,44,47,58,62,63,64,65,66].

In particular, it is known that specific components of pathogens such as bacteria, viruses, and fungi are recognized by toll-like receptors (TLRs) expressed by monocytes and macrophages: this binding results in the activation of intracellular signaling pathways leading to an increased expression of both VDR and CYP27B1 inside the cell, fostering cathelicidin and β-defensin 2 production [44,48,58,63,65,67,68].

**Figure 1:**
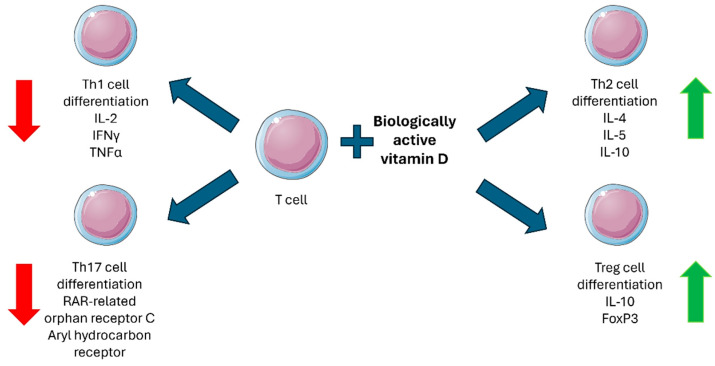
Biologically active vitamin D effects on T cell differentiation. Red arrows indicate a suppressive effect, and green arrows indicate a stimulatory effect. Figure was partly generated using Servier Medical Art, provided by Servier, licensed under a Creative Commons Attribution 4.0 unported license (https://creativecommons.org/licenses/by/4.0/).

Furthermore, VDR and CYP27B1, the key players in the immunomodulatory effects of vitamin D, are not exclusively expressed by monocytes/macrophages but are expressed in a tightly regulated manner also by lymphocytes. In particular, T cells are known to show an activation-state-dependent expression of VDR, while the biologically active vitamin D regulates their differentiation (Figure 1). Vitamin D is also known to modulate B cell functions in both an indirect and direct manner: while vitamin D-stimulated T helper cells suppress B cell differentiation and proliferation as well as antibody production, the biologically active form of the vitamin is also able to directly suppress naïve B lymphocyte differentiation towards plasma cells and memory B cells [21,44,58,62,63,64,65,66,68,69,70,71].

When considering immune defenses, the important role played by epithelial barriers should be highlighted, with the epithelium representing the first line of defense in many body districts, where it forms a physical barrier protecting the underlying visceral space from pathogen invasion. For this reason, epithelial barrier leakage due to direct pathogen interactions or the damaging effects secondary to pro-inflammatory cytokine production represents a critical event that could climax in multiorgan failure, especially in severely ill patients. Interestingly, epithelial cells express VDRs, thus representing a target for vitamin D endocrine signaling. Considering that epithelial barrier function relies on the junctional complexes connecting epithelial cells (mainly tight and adherent junctions) and that among the non-genomic biological effects of vitamin D, there is its ability to improve cell-to-cell junction’s functionality, it is not surprising that this vitamin could play an important role in improving host immunity also by acting at epithelial level [72,73,74]. Existing evidence supports the existence of a direct correlation between vitamin D deficiency and an increased risk of developing specific pathological conditions characterized by epithelial barrier compromise, especially at gastrointestinal (e.g., inflammatory bowel disease, celiac disease, ulcerative colitis, and Crohn’s disease) and pulmonary (e.g., chronic obstructive pulmonary disease, asthma, cystic fibrosis, and acute respiratory distress syndrome) level [73,74,75].

## 4. Vitamin D and Respiratory System Protection

Airways’ lumen is covered by a thin epithelial layer composed of specialized cells that closely interact with the immune cells harbored in the underlying connective tissue. Schematically, the airways’ epithelium is composed of a pseudostratified epithelial layer composed of different classes of cells connected to each other by different junctional complexes (tight and adherent junctions), forming a physical barrier between the inhaled air and the visceral space. The first line of defense assured by this physical barrier is assisted by the activity of ciliated and secretory primary cells in the epithelium, whose physiological role is to sustain mucociliary clearance of inhaled particulate and pathogens. Moreover, respiratory epithelial cells are also able to directly recognize invading pathogens through the toll-like receptor pathway and to respond accordingly by releasing antimicrobial peptides (e.g., defensins, cathelicidins, lysozyme, and lactoferrin) into the airway lumen as well as by releasing chemokines and cytokines (e.g., IL-1, TNF-α, IL-6, IL-8, eotaxin) into the submucosal layer to start an inflammatory response, which will activate immune response [76,77,78,79,80,81,82].

Interestingly, as previously stated, not only immune cells, but also respiratory epithelial cells express both VDR and CYP27B1 enzyme, making this anatomical district sensitive to vitamin D’s immunomodulatory activities [46,48,63,67,71,76,83]. Focusing on the respiratory system, it is known that airway epithelial cells are able to constitutively produce the active form of vitamin D and to increase its synthetic rate in response to viral infections. On the contrary, alveolar macrophages and dendritic cells only produce active vitamin D following pathogenic stimulation. In all cases, active vitamin D, by acting in an autocrine manner, plays a crucial role in fighting infectious agents [83,84].

From a mechanistic point of view, in vitro studies demonstrated that vitamin D is able, following an infection, to downregulate proinflammatory chemokine production in respiratory epithelium, as well as to support lymphocytes’ anti-inflammatory responses by modulating NF-κB signaling pathway [51,69,70,85]. Furthermore, in vitro studies also showed vitamin D’s ability to improve lung epithelial tight junctions’ functionality, by acting both on the proinflammatory milieu (mainly by reducing TNF-α levels) and on the junctional complex itself (mainly by reducing claudin-2 levels), thus representing a promising prophylactic measure against respiratory pathogens [86].

Since vitamin D exerts its biological roles by interacting with a specific receptor, also VDRs’ role in maintaining respiratory epithelial barrier integrity has been investigated. Animal studies in murine models demonstrated that VDR partial knockout resulted in a more severe LPS-induced pulmonary epithelial barrier damage, associated with reduced occludin and zonula occludens protein-1 expression. As already observed in in-vitro models, also in this case vitamin D was able to partially revert the damage, improving epithelial barrier function [73]. The key role of VDR in assuring pulmonary barrier functionality is even more evident when analyzing the effect of its complete knockout: VDR null mice showed an altered expression of tight and adherent junction-specific molecules, such as claudins, occludin, and zonula occludens protein-1, resulting in increased leakage of the epithelial barrier in pulmonary diseases [87,88].

According to both in vitro and animal models-derived evidence, the mechanisms leading to respiratory barrier dysfunction following infection and/or inflammation represent a multifactorial process, finally resulting in lung damage. Epithelial barrier damage is not only the direct result of pathogen interactions but can also be secondary to proinflammatory cytokines directly secreted by epithelial and endothelial cells. In both cases, vitamin D emerges as a potential resource to improve airway defense mechanisms [89].

The available literature on vitamin D’s involvement in pulmonary pathology highlights the correlation between hypovitaminosis D and inflammatory airway diseases, supporting its immunomodulatory and protective role, thus fostering its use as an add-on to the standard of care therapy to improve patient’s quality of life. In particular, recent intervention studies demonstrated vitamin D supplementation’s ability to reduce chronic obstructive pulmonary disease and asthma exacerbations, as well as viral and bacterial infections secondary to these pathological conditions, by improving respiratory epithelial barrier integrity, promoting antimicrobial peptides production, and modulating inflammatory and immune responses [85,90,91].

Furthermore, considering vitamin D’s involvement in lung physiology and the fact that the peak season of respiratory infections generally coincides with the lower circulating vitamin D levels, due to reduced sun exposure in winter months, it is not surprising that available literature shows an increased rate in respiratory infections in people with suboptimal vitamin D levels [45,46,53,62,66,70,89,92,93].

Recent studies revealed a relationship between hypovitaminosis D and tuberculosis prevalence, as well as the likelihood of disease progression from dormant to aggressive form [69,90]. Moreover, vitamin D supplementation in patients affected by tubercular infection has been shown to activate innate antibacterial as well as anti-inflammatory responses, thus representing a promising prophylactic approach in patients with latent infection [69,90,94].

Vitamin D is also known to protect against other respiratory infections. By improving cellular natural as well as adaptive immunity and epithelial barrier functions, it also protects individuals (and especially those already deficient of this nutrient) from the common cold and upper respiratory tract infections [90,95,96,97] as well as from more severe conditions such as acute lung injury and pneumonia [85].

However, existing meta-analyses and literature reviews of currently available controlled trials aimed to investigate vitamin D’s therapeutic role against airway infections yielded limited or inconclusive results [65,92,96,98,99,100,101,102], mainly because of the high heterogeneity in trial design, patients’ selection, vitamin D baseline levels, vitamin D supplementation posology, and endpoints definition. Nevertheless, the hypothesized prophylactic and protective role of this key nutrient in fighting respiratory infections is supported by some in vitro and animal studies, which cast new light on the still poorly understood underlying molecular mechanisms involved [49,65,70,75,89,100,102,103,104,105,106].

Considering these promising results against respiratory infections, it is not surprising that during the COVID-19 pandemic, vitamin D raised increasing interest as an add-on intervention to prevent or mitigate disease evolution.

## 5. Vitamin D and SARS-CoV-2 Infection

Despite the well-known extrapulmonary manifestations and complications of COVID-19, the disease is still primarily a respiratory infection. Respiratory epithelium lining the airways has been described as the primary infection site of SARS-CoV-2, a feature maintained by all the subsequent viral variants that have emerged so far, and which has evolved to improve viral fitness in such specific milieu in the more recent variants, such as Omicron BA.5 [107,108].

Since the beginning of the COVID-19 pandemic, several researchers have investigated SARS-CoV-2 infection and replication mechanisms, especially in the respiratory environment, revealing that viral spread and disease severity were strictly correlated with epithelium and immune cell interactions and that the most critical respiratory outcomes depend on hyperactivated immune responses finally resulting in exacerbated epithelial barrier destruction [109,110,111,112].

In the context of COVID-19 infection, respiratory epithelium plays a critical role, as it represents the primary SARS-CoV-2 target, due to the surface expression of ACE-2 and TMPRSS2, two key actors involved in viral entry, while acting as an immune organ involved in the neutralization of inhaled pathogens. Such a dual role of the airway epithelium accounts for its role in defining disease evolution, with the most severe clinical outcomes being generally associated with deep lung involvement [109,113].

Several research works cast new light on SARS-CoV-2 pathophysiologic mechanisms, further supporting the pivotal role of respiratory epithelium in viral systemic dissemination [114,115,116]. The first observation of a direct SARS-CoV-2 ability to damage epithelial barrier dates back to 2020 when Hao and coworkers [116] reported a decrease in transepithelial electrical resistance value along with the disappearance in zonula occludens-1 protein in a polarized human airway epithelium grown in an air–liquid interface system. More recently these results were confirmed and expanded by the work of Xu and colleagues [114], who described the role of viral protein E in airway barrier damage, highlighting its ability to down-regulate tight junctions’ integrity, by specifically targeting zonula occludens-1 protein, along with its ability to exacerbate inflammatory responses by increasing intracellular chloride concentrations. Furthermore, it should be considered that after airway infection, leukocytes, and particularly neutrophils, represent the first immune cells recruited to the site, where they start releasing pro-inflammatory mediators responsible for innate immune responses. Respiratory neutrophilia is a common feature of many chronic inflammatory lung diseases, and in the case of COVID-19, it has been correlated to disease severity. To better understand neutrophils’ role in fighting SARS-CoV-2 infection, Calvert and coworkers [115] developed an in vitro model of neutrophilic airways, showing that these granulocytes behave differently in the apical and basolateral compartment of the infected respiratory epithelium, as demonstrated by the specific pro-inflammatory cytokine profile observed, and that neutrophilia is responsible for the reduction in epithelial barrier integrity, thus supporting an increased viral spreading, due to an augmented translocation of the infecting virus from the apical to basolateral compartment of the epithelium.

The above-summarized evidence about SARS-CoV-2 pathophysiology highlights the critical role of airway epithelium and immune system crosstalk in supporting viral spread and dissemination. Furthermore, as respiratory epithelium activation can result in both type 1 and type 2 cytokine production and considering that these immune responses appear to be differently regulated according to disease prognosis [109], it is not surprising that vitamin D, which is known to exert an immunomodulatory action both at systemic and pulmonary level, has been investigated as a potential therapeutic and/or supportive intervention able to improve COVID-19 clinical outcomes.

Based on the previous observation that reduced vitamin D is a clinical condition that could result in increased susceptibility to upper respiratory tract infections [47,57,102,117], similar evaluations have been carried out also in the context of COVID-19 and, even if some conflicting results emerged, many studies highlighted a relationship between baseline vitamin D status and SARS-CoV-2 infection risk and/or severe disease outcomes [4,43,47,55,56,58,118,119,120,121,122,123]. Furthermore, an inverse relationship has been observed between subsequent disease wave peaks and solar irradiation, a key event in determining vitamin D production in the human body [30,124,125].

From a pathobiological point of view, it is well accepted that SARS-CoV-2 infection elicits proinflammatory cytokine release, which could heavily impact disease evolution. Given that vitamin D is known to counteract, at least partially, such phenomenon and that it is involved both directly and indirectly in the regulation of different thrombotic pathways, it is conceivable that great attention has been granted to vitamin D supplementation in order, not only to prevent viral infection, but also to reduce both ARDS risk and coagulation abnormalities, especially in severely ill patients [55,118,126,127].

The growing interest in the vitamin D’s supportive role in fighting COVID-19 is based on the increasing knowledge about its molecular actions and mechanistic interactions with ACE-2, one of the key players in disease evolution. ACE-2, which is known as the primary entry receptor for SARS-CoV-2, is one of the components of the renin-angiotensin-aldosterone (RAAS) system, an important endocrine axis involved in homeostasis maintenance. In particular, ACE-2 is responsible for the conversion of angiotensin II (a proinflammatory and pro-coagulant mediator) into angiotensin_(1–7)_ (an anti-inflammatory mediator). SARS-CoV-2 infection thus results in a depletion of ACE-2 with consequent increase of angiotensinogen II levels, which contribute to the observed sustained inflammation associated with COVID-19 evolution [126,128]. Among vitamin D’s biological effects, it is worth noting its ability to increase both the expression and bioavailability of ACE-2, both in its membrane-associated and soluble form, thus dampening the RAAS system, whose up-regulation is involved in cytokine storm generation [47,118,126]. From a mechanistic point of view, vitamin D’s protective effects mainly depend on its ability to inhibit renin, to increase angiotensin_(1–7)_ levels, and to increase ACE-2 bioavailability. In particular, vitamin D inhibits renin gene transcription, thus reducing RAAS axis activity and angiotensin II levels, while increasing angiotensin_(1–7)_, which has an important role in counterbalancing proinflammatory and pro-thrombogenic angiotensin II effect, by reducing TNF-α and IL6 expression and stimulating nitric oxide release from platelets, finally resulting in inflammation and coagulation abnormalities mitigation [47,58,126,128,129,130]. Furthermore, as previously stated, vitamin D is able to increase soluble ACE-2 that, contrary to the membrane-located form, has been demonstrated to act as a decoy viral receptor in early-stage infection, thus being involved in viral neutralization. In fact, soluble ACE-2 can bind SARS-CoV-2 viral particles, thus preventing them from reaching the membrane-bound form, and the resulting soluble ACE-2/SARS-CoV-2 complex are transported to natural killer cells and macrophages for destruction [128,131,132].

The large amount of possible positive effects of vitamin D in the contest of SARS-CoV-2 infection has fostered the design of several clinical trials aimed at the definition of clear clinical guidelines about its supplementation in COVID-19 patients. Unfortunately, as for many other dietary supplements tested as adjuvants in disease management, clinical research on vitamin D supplementation in COVID-19 patients led to mixed results, with some studies reporting beneficial effects, while others did not show any significant improvement in clinical outcomes [133,134,135,136,137]. As summarized in Table 2 [138,139,140,141,142,143,144,145,146,147,148,149,150,151,152,153,154,155,156,157,158], several studies reported an improvement in patients’ conditions after vitamin D supplementation [139,140,143,144,145,146,148,149,150,152,153,154,156,157,158], while others did not support the clinical adoption of such supportive intervention [138,141,142,147,151,155]. These inconclusive results mainly depend on the study design, showing a large heterogeneity in terms of vitamin D dose, formulation (biologically active vs. inactive metabolite), frequency and duration of the intervention (single bolus vs. continuous administration over longer periods) as well as in terms of population (i.e., age and disease severity, comorbidities, vitamin D basal status, hospital or outpatient setting).

In spite of the large literature evidence about the use of vitamin D supplementation in the context of COVID-19, the vast heterogeneity of the obtained results limited their generalizability. As shown in Table 2, several groups obtained promising results by integrating vitamin D into their standard-of-care routine approaches. Nevertheless, each group adopted different selection criteria, endpoints, doses, formulations, and posology, preventing the definition of clear guidelines about its clinical use. The increasing interest in such cheap and easy-to-use intervention, which could be of great support, especially in those situations where the available prophylactic and/or therapeutic resources are limited, highlights the need for continuous research on the topic. In particular, it is evident that novel clinical trials, designed adopting more standardized selection criteria and clinical as well as laboratory endpoints, showing sufficient statistical power to reach reliable results, will be essential to support national and international agencies in defining clear instructions, in terms of dose, formulation, and administration schedule to support clinicians in the choice of the most effective therapeutic approach for patients suffering from COVID-19 and, potentially, other related respiratory infections.

## 6. Vitamin D and Anti-SARS-CoV-2 Vaccination Response

The COVID-19 pandemic resulted in a wide effort from the pharmacological industry in developing new vaccines and, since 2020, more than 50 vaccines have been developed and tested in clinical trials (phase II and phase III). Thanks to this quickly evolving scenario, at the beginning of the worldwide mass vaccination campaign, several newly developed vaccines were approved by the competent international authorities [13,159,160,161].

In western countries, the vaccination campaign initially relied on two different vaccine platforms: the one based on recombinant messenger ribonucleic acid (mRNA), represented by the BNT162b2 (Pfizer-BioNTech, Mainz, Germany) and the mRNA-1273 (Moderna, Cambridge, MA, USA) vaccines, and the other based on partially inactivated adenovirus vectors, represented by the ChAdOx1-S (Astra-Zeneca, University of Oxford, UK) and the Ad.26.COV2.S (Janssen/Johnson & Johnson, New Brunswick, NJ, USA) [12,161]. Even if in recent years newly developed vaccine platforms became available, the most widely used worldwide is still represented by the mRNA-based one [11,159,162].

Although the initial clinical trials resulted in a high level of effectiveness of these vaccines [13,162,163,164,165,166], variations in responsiveness between recipients emerged from real-world data [160,167], thus fostering research focused on the understanding of the factors affecting vaccine efficacy, in order to improve world population protection against COVID-19 and reduce SARS-CoV-2 spread.

Considering its well-accepted immunomodulatory role and its easy availability as a nutritional supplement, vitamin D has gained attention also in this field. Previous evidence highlighted the effectiveness of vitamin D supplementation in inducing higher titers of specific IgG antibodies and improving mucosal immunity in response to different vaccines, such as those against tetanus, hepatitis B, and polio, both in animal models and humans [168,169,170,171,172].

To date, the investigation of vitamin D’s role in modulating immune response to the COVID-19 vaccine led to mixed results [159,167,173,174,175,176,177,178,179,180,181,182]. These conflicting results mainly depend on the vaccine platform (mRNA vs. adenoviral vectors), number of doses of the vaccine received, basal vitamin D status, vitamin D supplementation, comorbidities, previous exposure to the virus, and timing of IgG titer evaluation (peak response vs. specific time points) [159,175]. The most relevant clinical studies dealing with vitamin D’s effect on immune response after COVID-19 vaccination are summarized in Table 3.

As summarized in Table 3, results about vitamin D’s effects on anti-SARS-CoV-2 vaccine-elicited immune protection are conflicting, thus fostering further studies on the topic. From a mechanistic point of view, it could be hypothesized that, thanks to its well-known immunomodulatory activities, vitamin D could influence vaccine effectiveness by activating specific genetic pathways resulting in an enhanced T cell activation, proliferation, and conversion in memory T cells. Even if the main role of vitamin D in improving vaccine response seems to be related to its effects on T cell-mediated immunity, it can not be excluded also a possible role of this nutrient also in regulating B cell-mediated responses. Considering that an improvement of vaccine-elicited immune response could be important for all vaccinations, elucidating its mechanism of action could help to define clinical guidelines regarding vitamin D repletion before the beginning of the vaccination schedule, in order to obtain the maximum protection of the recipient.

## 7. Conclusions

Bibliographic research about the role of vitamin D in the context of COVID-19 supports the constant interest in the topic. Since the beginning of the pandemic, evidence accumulated regarding the possible relationship between vitamin D status and disease evolution. Ecological studies highlighted some interesting correlations between vitamin D levels and COVID-19: subsequent disease wave peaks have been shown to inversely correlate with solar radiation intensity and consequent vitamin D production, both in terms of timing and latitude. Moreover, disease morbidity and mortality have been shown to be related to the mean vitamin D status in different ethnic groups as, according to different authors, hypovitaminosis D differently affects populations with different skin pigmentation. Lastly, COVID-19’s most severe manifestations depend on hypercytokinemia and dysregulated immune response, two clinical features that could be influenced by vitamin D status. Considering the still accumulating evidence about the role of vitamin D as a modifiable risk factor for COVID-19 infection and/or negative outcomes, it is conceivable that the correction of hypovitaminosis D might act synergistically with the available therapies to improve clinical evolution, as well as with the anti-SARS-CoV-2 vaccines to improve immunological protection against infection.

To date, the mechanism by which vitamin D modulates COVID-19 evolution is still unknown: it appears to be reasonable that vitamin D’s protective effects might rely on different biological responses, such as antimicrobial peptides production, reduction in inflammatory mediator levels, and modulation of the renin-angiotensin pathway. For this reason, well-designed clinical trials are still needed to improve the understanding of vitamin D’s role in COVID-19 disease evolution and to establish evidence-based clinical guidelines for vitamin D supplementation, especially in populations at increased risk of developing more severe disease manifestations. Finally, a significant increase in vitamin D levels has been observed compared to the pre-pandemic period, mainly as a result of an uncontrolled, self-prescribed use of vitamin supplements. In particular, such rise in basal vitamin D status in the general population has been described to be temporarily associated with the emergence of widespread reports, often lacking rigorous scientific verification, claiming vitamin D’s benefits in COVID-19 management [183], thus further supporting the role of evidence-based information dissemination in ensuring patient safety, especially during public health emergencies.

## Figures and Tables

**Table 1:** Summary of selected recent clinical studies investigating nutraceutical over-the-counter solutions’ effectiveness in preventing or mitigating COVID-19 outcomes.

Over-the-Counter Solution	Country	Study Design	Main Findings	References
Lactoferrin	Italy	Multicenter, not-for-profit, randomized, double-blind, placebo-controlled, parallel-arm clinical trial	Lactoferrin, used as an adjuvant to the standard of care treatment, did not improve either clinical evolution or laboratory inflammation markers evaluation in moderate-to-severe COVID-19 patients	[31]
	Egypt	Single-center, randomized, prospective, interventional clinical trial	Lactoferrin, used as an adjuvant to the standard of care treatment, did not result in a statistically significant difference either in clinical resolution of the symptoms or in laboratory parameters	[32]
Vitamin C	India	Single-center, randomized, double-blind, placebo-controlled clinical trial	Vitamin C, used as an adjuvant to the standard of care treatment, reduced in-hospital mortality, need for mechanical ventilation, qSOFA index, inflammatory biomarkers, and need for vasopressors; moreover, it improved respiratory index (pO_2_/FiO_2_), even if the statistical significance was not reached due to the limited number of patients enrolled	[33]
	Iran	Single-center, randomized, double-blind, placebo-controlled clinical trial	High-dose intravenous vitamin C, used as an adjuvant to the standard of care treatment, did not statistically affect SOFA score or 28-day mortality compared to the standard of care alone	[34]
	Turkey	Retrospective study	High-dose intravenous vitamin C, used as an adjuvant to the standard of care treatment, did not statistically affect clinical outcomes in patients with severe COVID-19	[35]
	China	Retrospective cohort study	High-dose vitamin C, used as an adjuvant to the standard of care treatment, reduced the mortality and improved oxygen support status	[36]
	China	Multicenter, randomized, controlled clinical trial	High-dose intravenous vitamin C did not statistically affect invasive mechanical ventilation-free days in 28 days, but might have a potentially beneficial effect on oxygenation in critically ill patients, by improving PaO_2_/FiO_2_	[37]
	Iran	Single-center, randomized, open-label, controlled clinical trial	High-dose intravenous vitamin C, used as an adjuvant to the standard of care treatment, did not statistically affect clinical outcomes in patients with severe COVID-19	[38]
	USA	Multicenter, prospective, randomized, open-label clinical trial	Ascorbic acid, alone or in combination with high-dose zinc gluconate, did not significantly affect the symptoms’ duration compared to the standard of care therapy in an outpatient setting	[39]
Zinc	USA	Multicenter, prospective, randomized, open-label clinical trial	High-dose zinc gluconate, alone or in combination with ascorbic acid, did not significantly affect the symptoms’ duration compared to the standard of care therapy in an outpatient setting	[39]
Melatonin	Iran	Single-center, randomized, double-blind, clinical trial	High-dose melatonin, used as an adjuvant to the standard of care treatment for intubated patients reduced C-reactive protein levels but, apparently, had no effects on patient outcomes	[40]
	Iran	Single-center, randomized, double-blind, clinical trial	Melatonin, used as an adjuvant to the standard of care treatment, improved clinical symptoms (cough, dyspnea, fatigue), C-reactive protein and pulmonary involvement and, also, contributed to a faster return to baseline health	[41]
Omega 3 fatty acids	Mexico	Single-center, randomized, double-blind clinical trial	Omega 3 fatty acid supplementation reduced leukocyte counts and hematocrit levels, low-density lipoproteins and very low-density lipoproteins, glucose, creatinine, and blood urea nitrogen levels, while increasing high-density lipoprotein cholesterol in unvaccinated patients with moderate COVID-19	[24]
	China	Single-center, randomized, single-blind, group sequential, active-controlled clinical trial	Omega 3 fatty acid α-lipoic acid, used as an adjuvant to the standard of care treatment, reduced SOFA score and 30-day all-cause mortality with only borderline statistical significance, due to the limited number of patients enrolled	[42]
	Iran	Single-center, double-blind, randomized clinical trial	Omega 3 fatty acid enteral supplementation improved 1-month survival as well as several parameters of respiratory and renal functions (arterial pH, bicarbonate, base excess, creatinine, potassium and blood urea nitrogen) in critically ill COVID-19 patients	[25]

**Table 2:** Summary of the most relevant clinical trials investigating vitamin D (considering all the available pharmaceutical formulations) supplementation effectiveness in preventing or mitigating COVID-19 outcomes in adult patients.

Country	Study Design	Study Objective	Participants	Intervention	Main Findings	References
China	Multicenter, open-label, randomized controlled trial	Investigation of high-dose vitamin D_2_ ability to prevent COVID-19 and/or improve disease symptoms	248 health care workers were assessed for eligibility and 214 underwent randomization. Finally, 99 subjects allocated to the intervention arm received vitamin D_2_, while 103 subjects allocated to the non-intervention arm did not receive the intervention	200,000 IU vitamin D_2_ on the 1st and 14th day of the study (intervention arm) vs. no intervention	The provided intervention did not significantly prevent infection or improve COVID-19 symptoms. Nevertheless, it has been observed that there is a 14.3% difference in positive infection rates between patients with vitamin D levels > 30 ng/mL and patients with vitamin D levels < 20 ng/mL	[138]
Thailand	Prospective, open label, randomized controlled trial	Investigation of high-dose alfacalcidiol ability to improve clinical outcomes in patients with COVID-19 pneumonia	306 COVID-19 patients were assessed for eligibility and 294 underwent randomization. 147 subjects were allocated to the intervention group, and 147 subjects were allocated to the non-intervention group	2 µg daily or <0.05 µg/kg/day alfacalcidiol in addition to the standard treatment until discharge vs. standard treatment only	Vitamin D supplementation in addition to standard treatment was beneficial for patients with COVID-19 pneumonia who require supplemental oxygen or high-dose corticosteroid therapy or who show CRP levels > 30 mg/L upon treatment initiation	[139]
India	Single-center, randomized, double-bind, placebo-controlled clinical trial	Investigation of the effectiveness of a single oral high dose of cholecalciferol in improving sequential organ failure assessment (SOFA) score at 7 days, as well as the total duration of mechanical ventilation, all-cause mortality at 28 days and inflammatory marker levels	358 COVID-19 patients were assessed for eligibility and 90 underwent randomization. 45 subjects were allocated to the intervention arm, and 45 subjects were allocated to the placebo arm	60,000 IU cholecalciferol on the day of inclusion (intervention arm) in addition to the standard of care vs. placebo in addition to the standard of care	The administration of a high dose of cholecalciferol at the time of ICU admission improved SOFA score on day 7 and reduced in-hospital mortality in vitamin D-deficient COVID-19 patients	[140]
Croatia	Single-center, open-label, randomized clinical trial	Investigation of daily vitamin D supplementation ability to improve clinical outcomes in ICU patients	292 COVID-19 patients admitted to ICU were assessed for eligibility and 155 underwent randomization. 78 subjects were allocated to the intervention group (75 received vitamin D supplementation according to the study protocol) and 77 subjects were allocated to the non-intervention group	10,000 IU cholecalciferol administered daily for 14 days in addition to the standard of care vs. standard treatment alone	Vitamin D supplementation did not affect the number of days spent on respiratory support nor the prespecified secondary outcomes	[141]
Tunisia	Randomized controlled, parallel-group, blinded, clinical trial	Investigation of the effect of vitamin D supplementation on recovery delay among COVID-19 patients with positive RT-PCR at 14 days post-diagnosis confirmation	130 COVID-19 patients with positive RT-PCR 14 days after COVID-19 diagnosis confirmation were assessed for eligibility and 117 underwent randomization. 57 subjects were allocated to the intervention arm, and 60 subjects were allocated to the placebo arm	200,000 IU cholecalciferol vs. placebo	Vitamin D supplementation was not associated with a reduction in recovery delay when administered to patients with positive RT-PCR 14 days after COVID-19 diagnosis; furthermore, the median duration of RNA viral conversion was significantly longer in the intervention group compared to the placebo arm	[142]
Iran	Double-blinded, randomized clinical trial	Investigation of the effect of high and low dose vitamin D supplementation on liver function biomarkers in hospitalized COVID-19 patients	218 COVID-19 patients were assessed for eligibility and 140 underwent randomization. 70 subjects were allocated to the intervention arm (high dose vitamin D), and 70 subjects were allocated to the control arm (low dose vitamin D)	50,000 IU vitamin D at enrolment followed by 10,000 IU/mL daily till 30 days (high dose vitamin D group) vs. placebo (gelatin soft gel capsule) at enrolment followed by 1000 IU/mL vitamin D daily till 30 days (low dose vitamin D group)	High-dose vitamin D supplementation improved alkaline phosphatase markers in COVID-19 patients	[143]
USA	Multicenter, double-blinded, randomized, placebo-controlled phase 2 clinical trial	Investigation of the benefit of raising serum vitamin D to a level ≥ 50 ng/mL with an extended-release calcifediol (ERC) formulation on time to resolution of symptoms in COVID-19 patients in an outpatient setting	241 COVID-19 patients were assessed for eligibility and 171 underwent randomization. 85 subjects were allocated to the intervention arm, and 86 subjects were allocated to the placebo arm. 80 subjects for each group received at least one dose of the study drug	300 µg ERC for the first 3 days followed by 60 µg ERC from day 4 to day 27 vs. placebo	ERC treatment effectively increased serum vitamin D levels to the target level in mild-to-moderate COVID-19 patients in an outpatient setting and may have had a role in accelerating the resolution of respiratory symptoms, thus suggesting possible mitigation of COVID-19-related pneumonia risk	[144]
France	Multicenter, randomized, controlled, open-label, parallel-group, intent-to-treat, superiority clinical trial	Investigation of the ability of a single oral high dose of vitamin D_3_ administered within 72 h from COVID-19 diagnosis, compared to a standard vitamin D_3_ dose, to improve the 14-day overall survival in at-risk individuals	1027 COVID-19 elderly patients (>65 years) were assessed for eligibility and 260 underwent randomization. 130 subjects were allocated to the high-dose group, and 130 subjects were allocated to the standard-dose group. 126 subjects in the high dose and 127 subjects in the standard dose received the study drug	400,000 IU cholecalciferol on the day of inclusion (high dose group) vs. 50,000 IU cholecalciferol on the day of inclusion (standard dose group)	The early administration of a high dose of vitamin D_3_ to at-risk older patients suffering from COVID-19 improved the 14-day overall survival	[145]
Mexico	Multicenter, randomized, double-blind, parallel arm clinical trial	Investigation of the efficacy and safety of cholecalciferol supplementation in preventing SARS-CoV-2 infection in highly exposed individuals	407 healthcare workers were assessed for eligibility and 321 underwent randomization. 160 subjects were allocated to the intervention arm, and 161 subjects were allocated to the placebo arm. 150 (94 analyzed) subjects in the intervention arm and 152 (98 analyzed) subjects in the placebo arm received the study drug	4000 IU cholecalciferol for 30 days (intervention arm) vs. placebo	Vitamin D supplementation was effective and safe in preventing SARS-CoV-2 infection in highly exposed individuals regardless of the baseline vitamin D status	[146]
Spain, Argentina, Guatemala, Chile	Multicenter, randomized, controlled, open-label, international clinical trial	Investigation of the effectiveness of a high dose cholecalciferol administered at the time of hospital admission in modifying clinical outcomes in moderate-to-severe COVID-19 patients	570 COVID-19 patients were assessed for eligibility and 548 underwent randomization. 277 (274 analyzed) subjects were allocated to the intervention arm, and 271 (269 analyzed) subjects were allocated to the control arm	100,000 IU cholecalciferol on the day of inclusion (intervention arm) vs. no intervention (control arm)	The proposed intervention did not statistically improve clinical outcomes (reduction in median length of hospital stay, ICU admission rate, mortality rate)	[147]
India	Randomized, placebo-controlled clinical trial	Investigation of the effect of a high dose of cholecalciferol supplementation on SARS-CoV-2 viral clearance	89 COVID-19 patients were assessed for eligibility and 40 underwent randomization. 16 subjects were allocated to the intervention arm, and 24 subjects were allocated to the placebo arm	60,000 IU cholecalciferol for 7 days (intervention arm) vs. placebo for 7 days	The administration of a high dose of cholecalciferol helped in achieving viral clearance in a greater proportion of asymptomatic vitamin D-deficient individuals along with a significant decrease in fibrinogen	[148]
Russia	Randomized, single-center open-label clinical trial	Investigation of the effectiveness of vitamin D supplementation in modifying serum vitamin D level, complete blood count, CRP levels, and B cell subset on the 9th day of hospitalization compared to the first one.	311 COVID-19 patients were assessed for eligibility and 129 underwent randomization. 65 subjects were allocated to the intervention arm, and 64 subjects were allocated to the no-intervention arm. After serum vitamin D quantification, 56 subjects in the intervention arm received the study drug, while 54 subjects in the no-intervention arm received only the standard-of-care therapy	50,000 IU vitamin D on the 1st and 8th days of hospitalization in addition to the standard of care (intervention arm) vs. standard of care alone (no-intervention arm)	The proposed intervention resulted in an increase in serum vitamin D levels, neutrophil and lymphocyte counts, and a decrease in CRP levels in vitamin D-deficient patients. Moreover, vitamin D supplementation was associated with a reduction in CD38^++^CD27 transitional and CD27^−^CD38^+^ mature naïve B cells and an increase in CD27^−^CD38^−^ double negative B cells	[149]
Belgium	Single-center, randomized, double-blind, placebo-controlled clinical trial	Investigation of the ability of vitamin D supplementation to improve COVID-19 clinical outcomes in vitamin D deficient hospitalized patients	69 COVID-19 patients were assessed for eligibility and 50 underwent randomization. 26 subjects (21 analyzed) were allocated to the intervention arm, and 24 (22 analyzed) subjects were allocated to the placebo arm	25,000 IU cholecalciferol daily for the first 4 days and then once weekly for up to 6 weeks (for a maximum of 36 days; study exit corresponded to hospital discharge) in addition to the standard of care (intervention arm) vs. placebo daily for the first 4 days and then once weekly for up to 6 weeks in addition to the standard of care	The proposed intervention significantly reduced hospitalization length, and oxygen supplementation duration and improved patients’ clinical status (WHO scale)	[150]
Argentina	Multicenter, randomized, double-blind, sequential, placebo-controlled clinical trial * * The sequential design consisted of an adaptative design with two stages: stage 1—assessment of the effects of vitamin D supplementation on respiratory sepsis-related organ failure assessment (rSOFA); stage 2—assessment of the effects of vitamin D supplementation on clinical events. As per protocol, after the recruitment of the first 200 participants, a blind analysis was carried out and, based on that, the trial was terminated	Investigation of the ability of a single oral high dose of vitamin D_3_ to prevent respiratory worsening in hospitalized mild-to-moderate COVID-19 patients with risk factors for disease progression	256 COVID-19 patients were assessed for eligibility and 218 underwent randomization. 115 subjects were allocated to the intervention arm, and 103 subjects were allocated to the placebo arm	500,000 IU vitamin D_3_ on the day of inclusion (intervention arm) vs. placebo on the day of inclusion (placebo arm)	The proposed intervention did not prevent respiratory worsening in mild-to-moderate COVID-19 patients with risk factors for disease progression	[151]
Egypt	Single-center, prospective, randomized, controlled clinical trial	Investigation of the ability of a single oral high dose of cholecalciferol compared to a standard alfacalcidiol dose, to improve COVID-19 clinical evolution (improvement of oxygenation parameters, reduction in hospitalization length and mortality rate, variation in inflammatory markers, occurrence of secondary infections and adverse events) in hospitalized moderate-to-severe COVID-19 patients	116 COVID-19 patients were assessed for eligibility and underwent randomization. 58 subjects were allocated to the high-dose arm, and 58 subjects were allocated to the standard-dose arm	200,000 IU cholecalciferol on the day of inclusion in addition to the standard of care (high dose group) vs. 1 µg/day alfaclcidiol for at least 5 days in addition to the standard of care (standard dose group)	The high-dose cholecalciferol supplementation resulted in better clinical improvement and fewer adverse outcomes compared to the standard dose supplementation regimen in moderate-to-severe COVID-19 patients	[152]
Spain	Multicenter, single-blinded, prospective, randomized, clinical trial	Investigation of the safety, tolerability, and effectiveness of high dose in comparison with moderate dose vitamin D_3_ supplementation for 14 days in improving COVID-19 clinical evolution in hospitalized patients	87 COVID-19 patients were assessed for eligibility and 85 underwent randomization. 44 subjects were allocated to the high-dose arm, and 41 subjects were allocated to the moderate-dose arm	10,000 IU vitamin D_3_ in addition to the standard of care for 14 days (high dose group) vs. 2000 IU vitamin D_3_ in addition to the standard of care for 14 days (moderate dose group)	Both interventions were safe and did not cause significant adverse events. The high-dose supplementation was more effective in increasing serum vitamin D levels, especially in overweight and obese individuals. Moreover, the high-dose supplementation in addition to the standard of care was effective in improving oxygen requirements and in reducing hospitalization length in patients developing ARDS	[153]
USA	Multicenter, randomized, open-label clinical trial	Investigation of the effectiveness of calcitriol supplementation in improving clinical outcomes (need for oxygen supplementation, hospitalization length, need for ICU admission, mortality and readmission) in COVID-19 patients	50 consecutive COVID-19 patients underwent randomization. 25 subjects were allocated to the intervention arm, and 25 subjects were allocated to the no-intervention arm	0.5 µg calcitriol daily for 14 days or till hospital discharge, whichever occurred first, in addition to the standard of care vs. standard of care alone	The proposed intervention resulted in an improvement in oxygenation in COVID-19 patients, but did not result in a significant improvement in the other clinical parameters	[154]
Brazil	Multicenter, double blind, randomized, placebo-controlled clinical trial	Investigation of the ability of a single high dose of vitamin D_3_ to reduce the duration of hospital stay in patients with moderate or severe COVID-19	1240 COVID-19 patients were assessed for eligibility and 240 underwent randomization. 120 subjects were allocated to the intervention arm, and 120 subjects were allocated to the placebo arm. 117 subjects in the intervention arm and 118 subjects in the placebo arm received the study drug	200,000 IU vitamin D_3_ on the day of inclusion vs. placebo	High-dose vitamin D_3_ supplementation did not statistically reduce hospital stay length, the in-hospital mortality rate, the ICU admission rate, or the need for mechanical ventilation support	[155]
Saudi Arabia	Multicenter, randomized, open-label clinical trial	Investigation of the ability of a daily vitamin D_3_ supplementation for 2 weeks in reducing the time to symptoms recovery in mild-to-moderate COVID-19 patients	77 COVID-19 patients were assessed for eligibility and 73 underwent randomization. 35 subjects were allocated to the standard 1000 IU arm, and 38 subjects were allocated to the 5000 IU arm	5000 IU vitamin D_3_ daily for 2 weeks vs. 1000 IU vitamin D_3_ daily for 2 weeks	A 2-week supplementation with 5000 IU vitamin D_3_ was superior to a 2-week supplementation with 1000 IU vitamin D_3_ in resolving cough and ageusia in mild-to-moderate COVID-19 patients with sub-optimal vitamin D levels	[156]
Iran	Multicenter, randomized, double-blinded, placebo-controlled clinical trial	Investigation of the ability of a daily dose of 25-hydroxyvitamin D_3_ to increase circulating vitamin D levels in COVID-19 patients and to improve disease evolution (severity, hospitalization length, need for oxygen support, death rate, and lymphocyte count)	134 COVID-19 patients were assessed for eligibility and 106 underwent randomization. 53 subjects were allocated to the intervention arm, and 53 subjects were allocated to the placebo arm. 24 subjects in the intervention arm and 19 in the placebo arm completed the 2 months follow up	25 µg 25-hydroxyvitamin D_3_ daily for 60 days in addition to the standard of care (intervention arm) vs. placebo for 60 days in addition to the standard of care	The proposed intervention was effective in correcting vitamin D deficiency/insufficiency in COVID-19 patients, an effect that was associated with an increase in lymphocyte percentage and a decrease in neutrophil to lymphocyte ratio, a marker of improved clinical prognosis	[157]
Spain	Parallel-arm, pilot, open-label, randomized, double-masked clinical trial	Investigation of the effectiveness of early calcifediol administration in reducing ICU admission rate and mortality rate	76 COVID-19 patients were assessed for eligibility and underwent randomization. 50 subjects were allocated to the intervention arm, and 26 subjects were allocated to the control arm	0.532 mg calcifediol at admission, followed by 0.266 mg calcifediol on days 3 and 7 and then weekly until study exit (ICU admission, discharge, or death) in addition to the standard of care vs. standard of care alone	The proposed calcifediol supplementation schedule significantly reduced the ICU admission rate in hospitalized COVID-19 patients; furthermore, no deaths were recorded among the patients allocated to the intervention arm	[158]

**Table 3:** Summary of the most relevant clinical studies investigating vitamin D (considering all available pharmaceutical formulations) effects on immune response after COVID-19 vaccination (different vaccine platforms).

Country	Study Design	Vaccine Type	Main Findings	References
India	Open-label, placebo-controlled, interventional trial	ChAdOx1nCoV-19 (adenoviral vaccine)	Calcifediol supplementation improved the efficacy of ChAdOx1nCoV-19 vaccine by augmenting T cell activation, proliferation, and T cell memory responses	[173]
The Netherlands	Prospective observational cohort study	BNT162b2 and mRNA1273 vaccines (both mRNA vaccines)	No association was found between vitamin D concentrations and humoral or cellular immune response following vaccination with mRNA vaccine platforms	[174]
Jordan	Observational study	BNT162b2 vaccine (mRNA vaccine)	Baseline vitamin D levels had no effect on the short-term response to a single dose of BNT162b2 vaccine	[159]
Italy	Retrospective cohort study	BNT162b2 vaccine (mRNA vaccine)	Low baseline vitamin D levels negatively affected long-term humoral response to BNT162b2 dual vaccination	[175]
Belgium	Observational study (secondary analysis)	BNT162b2 vaccine (mRNA vaccine)	No significant differences in terms of binding (S1RBD IgG) or neutralizing (50% pseudovirus neutralization titer) response to BNT162b2 dual vaccination were observed neither in subjects with severe vitamin D deficiency (vitamin D levels < 20 ng/mL) compared to vitamin D sufficient subjects, nor in subjects under active vitamin D supplementation compared to non-supplemented subjects	[176]
Iraq	Multicenter randomized clinical trial	BNT162b2 vaccine (mRNA vaccine)	A daily 600 IU vitamin D_3_ supplementation for 14–16 weeks resulted in a significant increase in IgG levels after the second vaccination compared to the first one. Moreover, vitamin D_3_ supplementation also reduced the vaccine-associated side effects after the second dose administration compared to the first one	[177]
United Kingdom	Sub-studies nested within a randomized controlled trial	ChAdOx1nCoV-19 (adenoviral vaccine) and BNT162b2 (mRNA vaccine) vaccines	Vitamin D supplementation (800 IU/day or 3200 IU/day) did not influence IgG, IgA, and IgM anti-spike titers, neutralizing antibodies titers or IFN-γ concentrations in the supernatants of S peptide stimulated whole blood in adults with a sub-optimal vitamin D status at baseline	[178]
Italy	Observational, longitudinal, retrospective study	BNT162b2 vaccine (mRNA vaccine)	While there were no significant differences in baseline anti-spike IgG and T-cell responses according to vitamin D status, significant correlations emerged between baseline vitamin D levels and anti-S IgG and neutralizing antibodies titers at six months after the second vaccine dose	[179]
United Kingdom	Observational study	BNT162b2 vaccine (mRNA vaccine)	Baseline vitamin D positively affects vaccination response, as demonstrated by an average 29.3% greater peak of anti-spike IgG antibodies in subjects with serum vitamin D levels > 50 nmol/l	[180]
Greece	Observational study	BNT162b2 vaccine (mRNA vaccine)	Vitamin D levels showed a trend for a positive association with antibody titer after 3 months from the last vaccination dose (subjects with serum vitamin D in the range of 25.68–32.99 ng/mL showed a positive association with higher antibody titer when compared with subjects with serum vitamin D in the range of 4.1–18.99 ng/mL; no statistically significant results observed in subjects with serum vitamin D in the range of 18.9–25.67 and 33–69.8 ng/mL)	[167]
Romania	Observational study	BNT162b2 vaccine (mRNA vaccine)	Baseline vitamin D levels had no effect on antibody responses after BNT162b2 dual vaccination, except for a weak but significant correlation observed only among infection-naïve subjects younger than 60 years	[181]
Germany	Observational study	BNT162b2 vaccine (mRNA vaccine)	No significant differences in terms of IgG response to BNT162b2 dual vaccination were observed neither with respect to baseline vitamin D levels nor in relation to self-reported active vitamin D supplementation	[182]
